# Cerebral Amyloid Angiopathy-Related Inflammation: Report of a Case with Very Difficult Therapeutic Management

**DOI:** 10.1155/2015/483020

**Published:** 2015-08-16

**Authors:** Francesca Crosta, Berardino Orlandi, Federica De Santis, Gianni Passalacqua, Jacopo C. DiFrancesco, Fabrizio Piazza, Alessia Catalucci, Giovambattista Desideri, Carmine Marini

**Affiliations:** ^1^Geriatric Unit, Department of Life, Health & Environmental Sciences, University of L'Aquila, 67100 L'Aquila, Italy; ^2^Neurology Unit, Avezzano's Hospital, 67051 L'Aquila, Italy; ^3^Radiology Unit, Avezzano's Hospital, 67051 L'Aquila, Italy; ^4^Department of Neurology, San Gerardo Hospital, 20900 Monza, Italy; ^5^The Inflammatory Cerebral Amyloid Angiopathy and Alzheimer's Disease Biomarkers (iCA*β*) International Network, Department of Surgery and Translational Medicine, Milan Center for Neuroscience (NeuroMi), University of Milano-Bicocca, 20900 Monza, Italy; ^6^Radiology Unit, L'Aquila's Hospital, 67100 L'Aquila, Italy; ^7^Neurology Unit, Department of Life, Health & Environmental Sciences, University of L'Aquila, 67100 L'Aquila, Italy

## Abstract

*Background*. Cerebral amyloid angiopathy-related inflammation (CAA-ri) results from autoimmune response to beta-amyloid deposits in cerebral vessels. Its clinical course and complications have seldom been described in literature. *Case Report*. In a patient presenting with delirium and left hemiparesis the diagnosis of CAA-ri was supported by the finding of elevated anti-amyloid autoantibodies in the cerebrospinal fluid (CSF). Steroid therapy produced significant improvements in clinical and investigational assessments, but after two months, it caused Acute Respiratory Distress Syndrome. After steroid therapy discontinuation the patient presented a rapidly progressive dementia, Guillain-Barré syndrome, new cerebral ischemic lesions, and thrombosis of the right cephalic and subclavian veins that were treated with subcutaneous heparin. After a week the patient died because of brain hemorrhage. *Conclusion*. This case suggests caution in steroid therapy discontinuation and antithrombotic therapy administration in patients with CAA-ri. The CSF search of anti-amyloid autoantibodies could be helpful to support the diagnosis.

## 1. Introduction

Cerebral amyloid angiopathy-related inflammation (CAA-ri) represents the coexistence of cerebral amyloid angiopathy (CAA) and inflammation and is thought to be due to an autoimmune response to beta-amyloid deposits [1–4]. Cerebral spinal fluid (CSF) anti-amyloid-*β* autoantibodies have recently been demonstrated to be elevated during the acute phase of CAA-ri, returning to control level following clinical and radiological remission [4, 5]. Men and women are equally affected and the onset is generally in the seventh decade. Cognitive and behavioral changes are the most common symptoms, followed by focal neurological signs, headache, and seizures [3]. These clinical manifestations are associated with asymmetrical white matter lesions and with multiple cortical-subcortical microhemorrhages. CAA-ri is a reversible cause of progressive dementia but the pattern of cortical and subcortical symptoms, severity, and progression has not been extensively assessed in the available reports. Possible complications during the follow-up, including other autoimmune diseases, have seldom been reported. We describe a patient with CAA-ri who developed a severe cortical-subcortical dementia and serious complications, which affected his clinical course.

## 2. Case Report

An elderly man was admitted to our Intensive Neurological Unit in May 2013, because of the sudden onset of left hemiparesis and delirium. Neurological examination showed left hemiparesis, left homonymous hemianopia, dysarthria, spatial and temporal disorientation, sensory aphasia, and psychomotor slowness.

**Figure 1:**
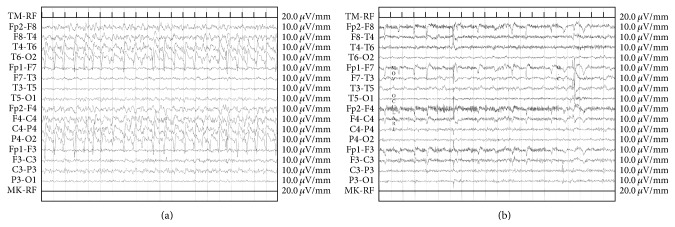
Electroencephalographic findings before (a) and after steroid therapy (b).

**Figure 2:**
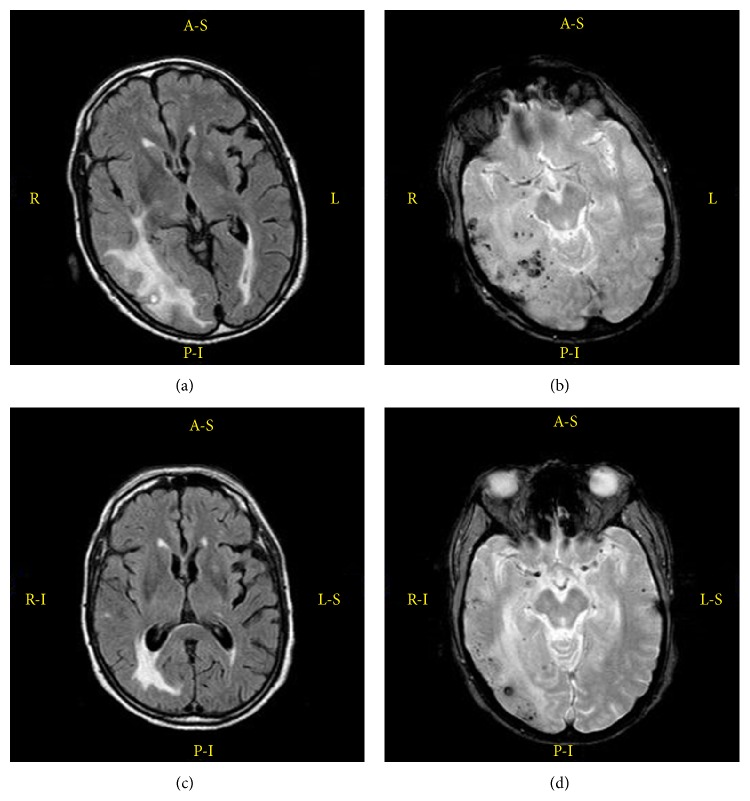
MRI findings of CAA-ri. Axial FLAIR images revealing bilateral hyperintense lesions (a). GRE images showing cortical-subcortical microhemorrhages (b). After one month of steroid therapy MRI sequences revealing the reduction of both cerebral edema and microhemorrhages (c-d).

CT scan showed hypodensity in the left occipital region and in the territory of the right middle cerebral artery with hyperdensities in its context along with compression of the right lateral ventricle. Blood tests revealed only an increased erythrocyte sedimentation rate (66 mm/h). Electroencephalogram (EEG) demonstrated the presence of subcontinuous 1-2 Hz delta waves and sharp-waves, which were more evident on the right side (Figure 1). MRI showed large white matter lesions in the right hemisphere and left occipital lobe on T2-weighted and fluid-attenuated inversion recovery (FLAIR) images suggesting vasogenic edema. Gradient-recalled echo (GRE) images revealed the presence of cortical and subcortical microhemorrhages in the white matter of temporal, parietal, and occipital lobes on the right side and of the occipital and parietal lobes of the left hemisphere (Figure 2). Cerebrospinal fluid (CSF) examination showed mildly elevated protein level and cell count and no evidence of infection. CAA-ri was suspected and the finding of APO-E genotype *ε*4/*ε*4 supported the diagnosis. Anti-amyloid *β* autoantibody concentration in CSF was 55.9 ng/mL. Physiotherapy and corticosteroid therapy with Dexamethasone 24 mg/day were started. After a few days, the patient presented a focal motor seizure on the left side and antiepileptic therapy was started. After one month, EEG was improved and showed a normal alpha-rhythm (Figure 1), while MRI showed a dramatic improvement of vasogenic edema together with less extensive and less definite microbleeding signals (Figure 2). Anti-amyloid *β* autoantibody concentration decreased to 49.9 ng/mL in CSF.

After twenty days, the patient was discharged home on steroid therapy. After two weeks, the patient was admitted to the Resuscitation Room because of the onset of Acute Respiratory Distress Syndrome (ARDS). Steroid therapy was discontinued. After one week, the patient was transferred to the Neurological Department. Cognitive performances had significantly worsened. MRI and EEG were unchanged. He presented distal muscle weakness, absent tendon reflexes, and progressive muscular atrophy. Electromyography confirmed the diagnosis of Guillain-Barré Syndrome (GBS). Intravenous immunoglobulin (0.6 g/kg) was started for five days with a slight improvement of patient's clinical conditions.

The cognitive assessment with Mental Deterioration Battery (MDB), performed after 2 months from symptom onset, revealed temporal disorientation, severe impairment of short-term memory and visual-spatial and executive functions, and lack of attention. Moreover, word-finding difficulties were pronounced and the content of language became vague and meaningless. The syntax of language output was poor (Table 1). After forty days of admission, the patient was discharged. Ten days later, the patient presented an acute loss of strength on his left upper limb and a worsening of cognitive status and functional skills. MDB revealed a worsening in orientation, attention, visual-spatial tasks, and executive functions (Table 1). Cogwheel rigidity, bradykinesia, and amimia were also identified, indicating the onset of Parkinsonism. A new MRI showed the presence of new cortical-subcortical ischemic lesion in right parietal and occipital regions, while EEG revealed the presence of 1-2 Hz delta waves and sharp-waves on the right side.

Steroid therapy was started again with Dexamethasone 4 mg twice a day, while antiplatelet agents were avoided. However, after a few days the patient presented a thrombosis of the right cephalic and subclavian veins. Thus subcutaneous Enoxaparin 4000 UI twice a day was started.

After one week the patient developed right hemiplegia and coma. CT scan showed a large intracerebral hematoma (diameter 8 × 6 cm) with associated perilesional edema, a midline shift of 2 cm, and subfalcal herniation. The same day the patient died.

## 3. Discussion

CAA-ri is a severe cerebrovascular disorder that affects elderly patients [4, 5]. The diagnosis may be made based on clinical data (subacute cognitive decline or seizures, headache, and encephalopathy), the presence of Apo-E *ε*4/*ε*4 genotype, response to steroid therapy, and MRI appearance (asymmetric vasogenic edema on T2 images and multiple microhemorrhages on GRE images) [3]. Nevertheless, CAA-ri is likely underdiagnosed because of its rare occurrence [6]. According to our experience, in the presence of delirium associated with stroke-like symptoms without adequate explanation on CT scan, MRI with GRE sequences should be performed. The detection of asymmetric white matter hyperintense signal on T2-weighted images and of multiple scattered cortical or subcortical microhemorrhages on GRE images is highly predictive of CAA-ri.

The differential diagnosis includes several clinical conditions. Posterior reversible encephalopathy syndrome (PRES) was excluded because of patient's age and clinical presentation, together with lack of predisposing conditions [7]. It has been also noted that patients with PRES and those with noninflammatory CAA have symmetric lesions, whereas patients with CAA-ri have asymmetric inflammatory lesions, as in our patient. Mitochondrial encephalopathy, lactic acidosis, and stroke-like syndrome were excluded because of clinical presentation and age of the patient.

The patient's clinical presentation, blood examinations, and lumbar puncture allowed us to exclude meningoencephalitis and vasculitis [6]. The response to steroid therapy and the demonstration of the APO-E *ε*4/*ε*4 genotype helped to clarify the diagnosis. Moreover, the identification of elevated anti-beta amyloid autoantibodies in the CSF furtherly supported the diagnosis. Recent studies have suggested that CSF levels of anti-beta amyloid antibodies may be a valid tool for the diagnosis of CAA-ri. The presence of high titer of these autoantibodies (above 32 ng/mL) in the CSF suggests the pathophysiologic mechanism of a selective autoimmune reaction against cerebrovascular beta-amyloid deposits [4, 5].

The cause for which this autoimmune response occurs is not well understood, although it is seen more frequently in patients with APO-E *ε*4/*ε*4 genotype [8].

Most cases of CAA-ri appear to be clinically monophasic when treated. Nevertheless relapses can occur [9]. In our case the relapse was attributed to early discontinuation of steroid therapy after one month, which was due to the onset of ARDS, even though the good clinical response might also have suggested steroid therapy tapering.

In our patient CAA-ri contributed to the development of rapidly progressive dementia characterized by a severe impairment of both cortical and subcortical functions. Although recent reports showed the association with Alzheimer's disease [10], CAA-ri is a reversible cause of dementia and an early diagnosis is thus fundamental. As already reported in the literature [4, 6], our patient showed a relevant improvement with steroid therapy and a subsequent impairment after treatment discontinuation, furtherly suggesting the efficacy of the therapy.

Parkinsonism, as a complication of CAA-ri, has not been reported in the literature. The onset of Parkinsonism might have been caused by widespread lacunar infarcts in the basal ganglia. Interestingly, in our patient treatment with Dopamine agonist had to be discontinued because of the onset of visual hallucinations.

The onset of stroke-like symptoms, Parkinsonism, and rapidly progressive dementia were also due to new cortical-subcortical ischemic lesions probably due to the occlusion in amyloid-laden vessels [6]. Although antithrombotic therapy should be taken into account to avoid ischemic lesions and complications related to immobilization, the high risk of intracerebral hemorrhage usually prevents its use.

Antiplatelet agents were avoided in our patient due to the risk of bleeding, even after MRI had showed the occurrence of new ischemic lesions. However, despite physiotherapy, the patient became definitively bedridden after the onset of ARDS, GBS, stroke, Parkinsonism, and dementia. Those disorders favored the onset of subclavian venous thrombosis.

After a thorough discussion on the risk and benefit ratio, despite literature suggestions, it was collegially decided to start subcutaneous Enoxaparin therapy.

Unfortunately, after a few days, the patient developed a very large intracerebral hemorrhage and died. In our opinion, the coexistence of amyloid angiopathy and autoimmune vasculitis produces a very increased risk of bleeding. Although we do not know the exact balance of risk and benefits in individual cases, in our opinion those therapies should be used with caution even in the presence of thrombotic complications.

## 4. Conclusion

In the present case, in which clinical diagnosis was confirmed by CSF finding of anti-beta amyloid antibodies, the careful follow-up showed that CAA-ri may produce a rapidly progressing though reversible dementia and Parkinsonism. Steroid therapy should not been discontinued too early in order to prevent relapses. Antithrombotic therapy should be avoided as far as possible due to the high risk of bleeding. However, in our patient the occurrence of pulmonary, autoimmune, and thrombotic complications required very difficult treatment choices and led to death.

## Figures and Tables

**Table 1:** Results of Mental Deterioration Battery.

Mental Deterioration Battery	Three months after the onset		Four months later	
	Raw score	Adjusted score	Raw score	Adjusted score
Temporal orientation	0	0	2	2
Spatial orientation	4	4	2	2
Attentional matrices	16	23.25	5	12.25
TMT-A	NE	NE	NE	NE
Rey's Auditory Verbal Learning immediate recall	16	26	16	26
Rey's Auditory Verbal Learning delayed recall	NE	NE	NE	NE
Copying drawings without landmarks	2	3.3	1	2.3
Copying drawings with landmarks	17	19.1	0	2.1
Phonological verbal fluency	5	14	3	12
Phrase construction	NE	NE	NE	NE
Raven's coloured matrices	NE	NE	NE	NE

TMT-A = Trail making test-A; NE = patient unable to perform cognitive tests.
